# Foot-and-Mouth Disease Virus Interserotypic Recombination in Superinfected Carrier Cattle

**DOI:** 10.3390/pathogens11060644

**Published:** 2022-06-03

**Authors:** Ian Fish, Carolina Stenfeldt, Edward Spinard, Gisselle N. Medina, Paul A. Azzinaro, Miranda R. Bertram, Lauren Holinka, George R. Smoliga, Ethan J. Hartwig, Teresa de los Santos, Jonathan Arzt

**Affiliations:** 1 Foreign Animal Disease Research Unit, Plum Island Animal Disease Center, Agricultural Research Service, United States Department of Agriculture, Greenport, NY 11957, USA; ian.fish@usda.gov (I.F.); carolina.stenfeldt@usda.gov (C.S.); edward.spinard@usda.gov (E.S.); gisselle.medina@usda.gov (G.N.M.); paul.azzinaro@usda.gov (P.A.A.); miranda.bertram@usda.gov (M.R.B.); lauren.holinka-patterson@uconn.edu (L.H.); george.smoliga@usda.gov (G.R.S.); ethan.hartwig@usda.gov (E.J.H.); teresa.delossantos@usda.gov (T.d.l.S.); 2 Department of Diagnostic Medicine/Pathobiology, College of Veterinary Medicine, Kansas State University, Manhattan, KS 66506, USA; 3 National Bio and Agro-Defense Facility (NBAF), Agricultural Research Service, United States Department of Agriculture, Manhattan, KS 66502, USA

**Keywords:** foot-and-mouth disease virus, aphthovirus, FMD, FMDV, pathogenesis, coinfection, recombination, persistent infection

## Abstract

Viral recombination contributes to the emergence of novel strains with the potential for altered host range, transmissibility, virulence, and immune evasion. For foot-and-mouth disease virus (FMDV), cell culture experiments and phylogenetic analyses of field samples have demonstrated the occurrence of recombination. However, the frequency of recombination and associated virus–host interactions within an infected host have not been determined. We have previously reported the detection of interserotypic recombinant FMDVs in oropharyngeal fluid (OPF) samples of 42% (5/12) of heterologously superinfected FMDV carrier cattle. The present investigation consists of a detailed analysis of the virus populations in these samples including identification and characterization of additional interserotypic minority recombinants. In every animal in which recombination was detected, recombinant viruses were identified in the OPF at the earliest sampling point after superinfection. Some recombinants remained dominant until the end of the experiment, whereas others were outcompeted by parental strains. Genomic analysis of detected recombinants suggests host immune pressure as a major driver of recombinant emergence as all recombinants had capsid-coding regions derived from the superinfecting virus to which the animals did not have detectable antibodies at the time of infection. In vitro analysis of a plaque-purified recombinant virus demonstrated a growth rate comparable to its parental precursors, and measurement of its specific infectivity suggested that the recombinant virus incurred no penalty in packaging its new chimeric genome. These findings have important implications for the potential role of persistently infected carriers in FMDV ecology and the emergence of novel strains.

## 1. Introduction

Foot-and-mouth disease virus (FMDV; genus Aphthovirus, family Picornaviridae), the causative agent of the eponymous disease, is a rapidly evolving pathogen that impacts livestock across much of the world. Currently, there are six antigenically distinct serotypes in circulation defined by the VP1 capsid-encoding region of the genome: A, O, Asia1, SAT 1, SAT 2, and SAT 3. Multiple serotypes regularly co-circulate in endemic regions and animals have been found to be naturally coinfected with multiple serotypes [1,2,3,4]. Further, FMDV has a high rate of mutation which contributes to a need for continuous development of updated vaccines and genomic surveillance.

The clinical phase of FMDV infection in naïve cattle is characterized by fever and vesiculo-erosive lesions in the mouth and on the feet [5]. Importantly, FMDV can persist subclinically in the upper respiratory tract of cattle for months to years [6,7,8]. These FMDV carriers are defined by detection of infectious virus in oropharyngeal fluid (OPF) sampled using a probang cup at least 28 days after initial infection [9,10]. Importantly, animals with natural or vaccine-induced immunity can become subclinically infected with FMDV, regardless of the occurrence of clinical FMD [7]. In cattle, both the early (neoteric) and persistent forms of subclinical infection are restricted to the nasopharyngeal mucosa [11]. The main distinction between these two phases of FMDV infection is that neoteric infection is associated with substantial virus shedding in oronasal secretions, whereas probang sampling is necessary to detect infectious virus during the persistent phase of infection [12,13].

Historically, laboratory investigations into FMDV recombination detected genomic crosses of conditionally lethal mutants in tissue culture [14,15,16,17]. Subsequently, the detection of recombination between distinct FMDV genomes has been largely limited to broad phylogenetic studies [18,19,20,21]. FMDV recombination in cattle under controlled experimental conditions has only been described in the predecessor to this current study, which reported that recombinant FMDVs were detected in 42% of superinfected carrier cattle [22]. The same study reported that no recombinant FMDVs were detected in samples from cattle simultaneously co-infected with distinct strains of FMDV.

The mechanism by which picornavirus recombination is believed to take place is termed ‘copy choice’ or ‘template switching’, and consists of the RNA-dependent RNA polymerase (RdRp) switching from one template genome to another during replication, thereby creating a hybrid product [23,24]. While recombinants from infection within the genome of a single FMDV strain would not be readily detectable, coinfection with two distinct serotypes can produce recombinants with detectable mosaic genomes. Patterns of recombination are shared between FMDV and other picornaviruses. The most common breakpoints, or recombination ‘hotspots’, are reported between the structural (capsid-encoding) regions and the non-structural protein coding regions [25,26,27]. For FMDV, though multiple breakpoints have been identified in all non-structural proteins as well as in the 5′-UTR, some more common hotspots are coding regions VP4, 2A, and 2C [17,19,25].

The present work builds upon the recently-published investigation that followed the clinical progression and infection dynamics of cattle coinfected with FMDV serotypes A and O [22]. Within a subset of coinfected cattle, interserotypic chimeric viruses were identified. Detailed analysis of these recombinant viruses and their associated populations is the focus of the present work.

## 2. Results

### 2.1. Animal Study and Virus Identity Results

**Figure 1:**
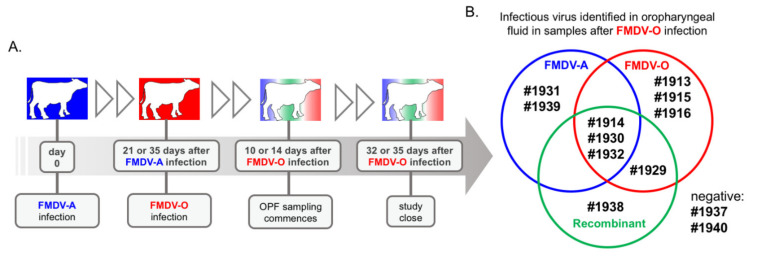
Study schematic and results overview. (**A**) A total of 12 cattle were infected with FMDV A24 Cruzeiro by intra-nasopharyngeal deposition on study day 0. On day 21 (eight cattle), or day 35 (four cattle), the animals were superinfected with FMDV O1 Manisa using the same route and dosage. Samples of oropharyngeal fluid (OPF) were obtained using a probang cup, starting at 10 or 14 days after superinfection and continued biweekly through to the termination of the studies at 32 or 35 days after superinfection. (**B**) Illumina sequencing of OPF samples revealed that distinct samples contained the parental viruses alone or combined with other viruses, including inter-serotypic recombinant viruses.

Virus samples used for the current investigation were derived from a set of experimental studies of FMDV coinfection in cattle [22]. Specifically, these viruses were isolated from oropharyngeal fluid sampled from 12 cattle that were persistently infected with FMDV A24 Cruzeiro (FMDV-A), and subsequently superinfected with FMDV O1 Manisa (FMDV-O). In brief, the animals were first inoculated with FMDV A24 Cruzeiro (serotype A) through intra-nasopharyngeal deposition. All cattle developed classical clinical signs of FMD, including fever and vesicles on the feet and mouth. Oropharyngeal fluid samples consistently contained infectious FMDV during the transitional (post-acute to persistent) phase of infection. After either 21 (eight cattle) or 35 (four cattle) days, animals were superinfected with FMDV O1 Manisa (serotype O) by intra-nasopharyngeal deposition (Figure 1). The incidence of clinical signs upon serotype O infection was variable in the 21-day coinfection group, where two animals developed full classical FMD and six animals were subclinically infected with FMDV-O, confirmed through virus isolation (VI). In the 35-day coinfection group, all cattle developed full classical FMD upon infection with serotype O.

Oropharyngeal fluid sampling was paused during the acute phase of infection to not interfere with disease pathogenesis. At 10 or 14 days after the serotype O superinfection, sampling of OPF was performed at 3–4 day intervals over the course of 14–28 days (Figure 1, Table 1). Through this period, all samples from two cattle, animal IDs #1937 and #1940, were VI-negative, indicating clearance of infection. Samples from animals #1913, #1915, #1916, #1931, and #1939 contained single non-recombinant FMDV-A or FMDV-O viruses (Figure 1). OPF samples from the remaining five cattle, IDs #1914, #1929, #1930, #1932, and #1938, were found to include interserotypic recombinant FMDV (Figure 1). To the authors’ knowledge, interserotypic FMDV has not previously been isolated from a host in which the recombination events took place. The prevalence (42% of superinfected cattle) and independent emergence of these recombinant viruses was remarkable.

### 2.2. Virus Population Sequencing and Characterization

In order to investigate the virus populations associated with the emergence of interserotypic recombinant FMDV, all VI-positive OPF samples isolated following superinfection with FMDV serotype O were Illumina-sequenced as previously described [22]. All samples were sequenced in duplicate, at minimum. Total reads mapped to FMDV-A and FMDV-O reference genomes ranged from 231,427–4,152,638 per sample (Table 1). Average coverages ranged from 3702–69,258 reads per site. Recombinant viruses were initially identified by analyzing site-wise coverage of mapped reads to each reference genome. Recombinants were identified by characteristic shifts in reference genome coverage, e.g., a 100-fold change in genome coverage of one reference paired with a commensurate opposite change in coverage of the other at homologous genomic loci (breakpoints). Paired-end reads were regularly broken at these breakpoints, with the forward read mapping to one reference and the reverse read to the other. This method of investigation also indicated that many of the samples included evidence of secondary (minority) recombinants (addressed below). Recombinants were also confirmed through targeted RT-PCR of regions crossing breakpoints followed by traditional Sanger sequencing [22].

**Figure 2:**
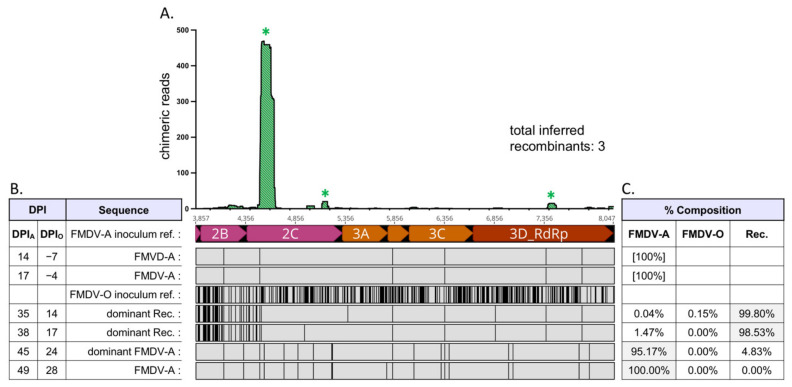
Serial FMDV sample sequencing analysis: Animal #1914. Foot-and-mouth disease viruses isolated from oropharyngeal fluid (OPF) samples were sequenced and analyzed. (**A**) Locations (*x*-axis) and counts (*y*-axis) of chimeric reads identified in OPF samples for coding regions 2A–3D; included sample timepoints ranged from 35 dpi_A_/14 dpi_O_ through 45 dpi_A_/24 dpi_O_. Asterisk (*) denotes multiple paired chimeric reads confirmed; as multiple breakpoints may exist on individual recombinant genomes, the total number of inferred recombinants is also noted. (**B**) Alignment of majority viruses identified at each time point in OPF samples, coding regions 2A–3D. The 5′ regions of recombinant viruses up to 2A were fully FMDV-O-derived. Serotype A is the reference for the alignment. Infection with serotype O occurred at 21 dpi_A_/0 dpi_O_. An FMDV-O reference sequence has been included for comparison. Abbreviations: dpi—days post-infection; Rec.—recombinant; ref.—reference. (**C**) Relative composition of viruses identified in each sample.

**Figure 3:**
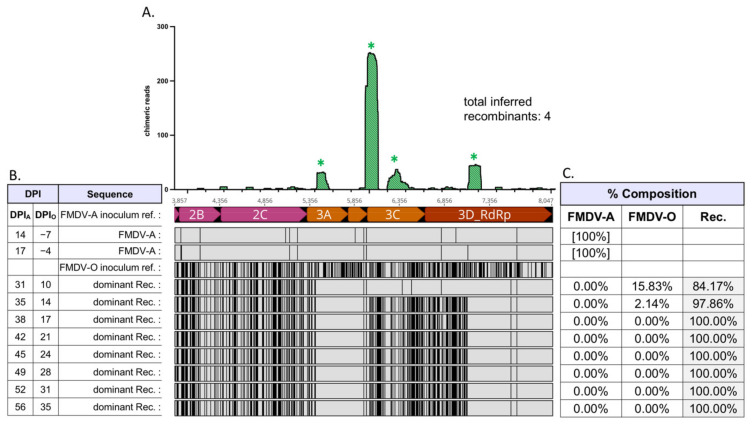
Serial FMDV sample sequencing analysis: Animal #1929. Foot-and-mouth disease viruses isolated from oropharyngeal fluid (OPF) samples were sequenced and analyzed. (**A**) Locations (*x*-axis) and counts (*y*-axis) of chimeric reads identified in OPF samples for coding regions; included sample timepoints ranged from 31 dpi_A_/10 dpi_O_ through 52 dpi_A_/31 dpi_O_. Asterisk (*) denotes multiple paired chimeric reads confirmed; as multiple breakpoints may exist on individual recombinant genomes, the total number of inferred recombinants is also noted. (**B**) Alignment of majority viruses identified at each time point in OPF samples, coding regions 2A–3D. The 5′ regions of recombinant viruses up to 2A were fully FMDV-O-derived. Serotype A is the reference for the alignment. Infection with serotype O occurred at 21 dpi_A_/0 dpi_O_. An O reference sequence has been included for comparison. Abbreviations: dpi—days post-infection; Rec.—recombinant; ref.—reference. (**C**) Relative composition of viruses identified in each sample.

**Figure 4:**
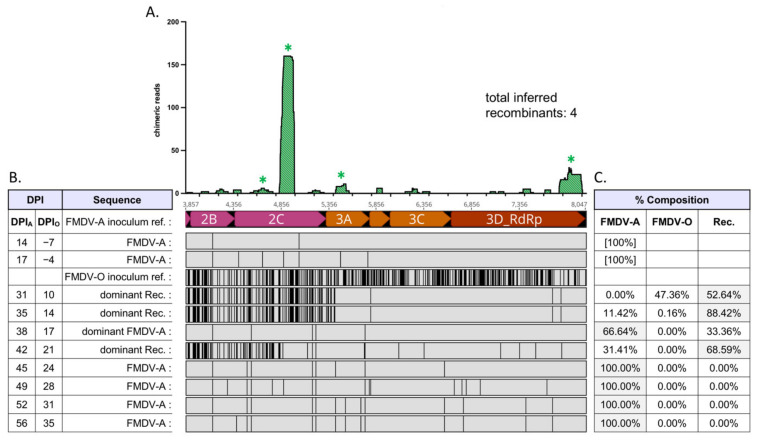
Serial FMDV sample sequencing analysis: Animal #1930. Foot-and-mouth disease viruses isolated from oropharyngeal fluid (OPF) samples were sequenced and analyzed. (**A**) Locations (*x*-axis) and counts (*y*-axis) of chimeric reads identified in OPF samples for coding regions 2A–3D; included sample timepoints ranged from 31 dpi_A_/10 dpi_O_ through 42 dpi_A_/21 dpi_O_. Asterisk (*) denotes multiple paired chimeric reads confirmed; as multiple breakpoints may exist on individual recombinant genomes, the total number of inferred recombinants is also noted. (**B**) Alignment of majority viruses identified at each time point in OPF samples, coding regions 2A–3D. The 5′ regions of recombinant viruses up to 2A were fully FMDV-O-derived. Serotype A is the reference for the alignment. Infection with serotype O occurred at 21 dpi_A_/0 dpi_O_. An FMDV-O reference sequence has been included for comparison. Abbreviations: dpi—days post-infection; Rec.—recombinant; ref.—reference. (**C**) Relative composition of viruses identified in each sample.

**Figure 5:**
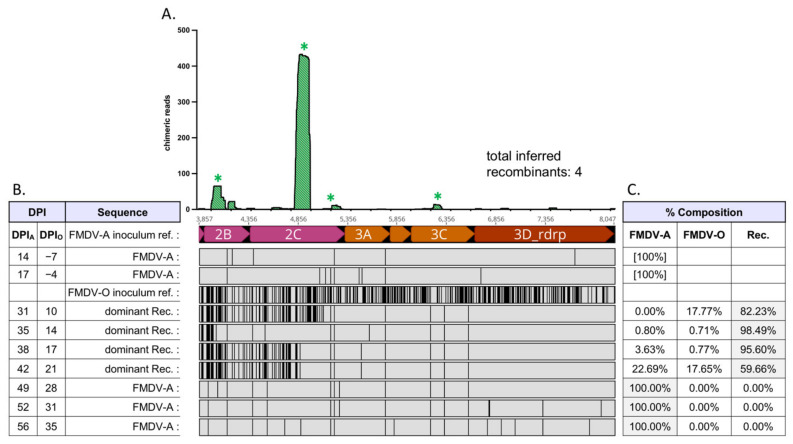
Serial FMDV sample sequencing analysis: Animal #1932. Foot-and-mouth disease viruses isolated from oropharyngeal fluid (OPF) samples were sequenced and analyzed. (**A**) Locations (*x*-axis) and counts (*y*-axis) of chimeric reads identified in OPF samples for coding regions 2A–3D; included sample timepoints ranged from 31 dpi_A_/10 dpi_O_ through 42 dpi_A_/21 dpi_O_. Asterisk (*) denotes multiple paired chimeric reads confirmed; as multiple breakpoints may exist on individual recombinant genomes, the total number of inferred recombinants is also noted. (**B**) Alignment of majority viruses identified at each time point in OPF samples, coding regions 2A–3D. The 5′ regions of recombinant viruses up to 2A were fully FMDV-O-derived. Serotype A is the reference for the alignment. Infection with serotype O occurred at 21 dpi_A_/0 dpi_O_. An FMDV-O reference sequence has been included for comparison. Abbreviations: dpi—days post-infection; Rec.—recombinant; ref.—reference. (**C**) Relative composition of viruses identified in each sample.

**Figure 6:**
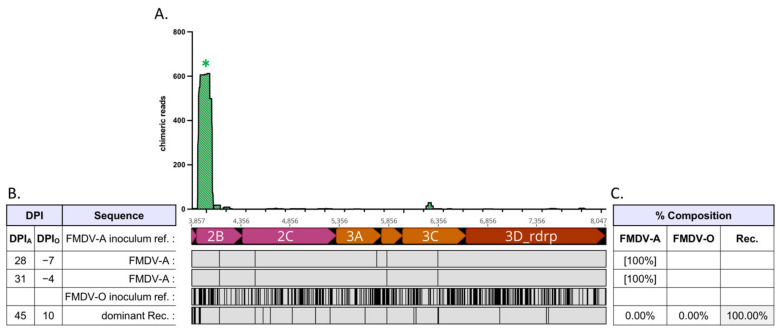
Serial FMDV sample sequencing analysis: Animal #1938. Foot-and-mouth disease viruses isolated from oropharyngeal fluid (OPF) samples were sequenced and analyzed. (**A**) Locations (*x*-axis) and counts (*y*-axis) of chimeric reads identified in the 45 dpi_A_/24 dpi_O_ OPF sample for coding regions 2A–3D. Asterisk (*) denotes multiple paired chimeric reads confirmed. (**B**) Alignment of majority viruses identified at each time point in OPF samples, coding regions 2A–3D; OPF sampled 14–24 dpi_A_/-(21-11) dpi_O_ are not shown, but were identical to 31 dpi_A_/-4 dpi_O_ within the 2A–3D region. The 5′ regions of the recombinant virus up to 2A was fully FMDV-O-derived. Serotype A is the reference for the alignment. Infection with serotype O occurred at 35 dpi_A_/0 dpi_O_. An FMDV-O reference sequence has been included for comparison. Abbreviations: dpi—days post-infection; Rec.—recombinant; ref.—reference. (**C**) Relative composition of viruses identified in each sample.

Virus populations present in OPF samples of superinfected cattle were composed of either non-recombinant (parental) FMDV-A, non-recombinant (parental) FMDV-O, and/or interserotypic recombinant viruses (Figure 1, Table 1). Relative frequencies of FMDV-A, FMDV-O, and (collective) interserotypic recombinant genomes were calculated based on reference genome coverage (Figure 2C, Figure 3C, Figure 4C, Figure 5C and Figure 6C). Recombinant viruses were found to be the most abundant (dominant) virus in at least one OPF sample from each of the five cattle in which recombinants were identified (Figure 2C, Figure 3C, Figure 4C, Figure 5C and Figure 6C, Table 1). Additionally, the first date of OPF sampling following superinfection at 31 days post-infection with FMDV-A (dpi_A_)/10 days post-infection with FMDV-O (dpi_O_) or 35 dpi_A_/14 dpi_O_ was also the first observation of recombinant viruses for all animals in which recombinants were detected. Importantly, these timepoints fell within the early (neoteric) phase of the second FMDV infection, when transmission remains viable [28]. Further, viruses isolated from nasopharyngeal tissue samples contained recombinant viruses as early as 48 h after FMDV-O infection (data not shown). For animals #1914, #1930, and #1932, recombinants comprised the majority virus in OPF samples for at least two sampling dates spanning at least 4 days (Figure 2, Figure 4 and Figure 5). Samples obtained from animal #1938 included only one VI-positive OPF with evidence of only a single FMDV species (a recombinant) at 10 days after serotype O infection (Figure 6). Viruses detected in samples from animal #1929 were exclusively recombinant from 38 dpi_A_/17 dpi_O_ through study close at 56 dpi_A_/35 dpi_O_ (Figure 3). The same approach was applied to numerous acute (vesicle) samples with no identification of recombinant viruses.

Chimeric reads were defined as reads composed of both FMDV-A-derived and FMDV-O-derived sequence. Chimeric read data from OPF samples for each animal were combined and plotted along an *x*-axis representing coding region 2A–3D, which is homologous (i.e., each protein coding region is the same length) between both parental serotypes (Figure 2, Figure 3, Figure 4, Figure 5 and Figure 6). Subconsensus analysis indicated that most of the earlier OPF samples (e.g., 31 dpi_A_/10 dpi_O_ and 35 dpi_A_/14 dpi_O_) included multiple interserotypic recombinants. Minority recombinants identified commonly became dominant in the following OPF samples. For example, a minority recombinant virus in the #1929 52 dpi_A_/31 dpi_O_ sample was identified as the majority recombinant virus in the subsequent 56 dpi_A_/35 dpi_O_ sample (Figure 3). All recombinants shared a similar genomic structure; while the specific breakpoints were variable, all recombinant genomes were derived from the FMDV-O parental genome 5′ of coding region 2B (Figure 2, Figure 3, Figure 4, Figure 5 and Figure 6, alignments). Thus, the 5′-UTR, leader protease, and all FMDV structural proteins (capsid-encoding regions) were derived from the FMDV-O inoculum. Chimeric reads belonging to recombinants that remained at minority levels were also consistently composed of FMDV-O-derived 5′-oriented segments and FMDV-A-derived 3′-oriented segments. The only exception to this was those chimeric reads associated with the multiple-recombinant in #1929, specifically in the 3B–3C region. Dominant recombinants had diverse breakpoints across the nonstructural coding regions including 2B, 2C, 3A, 3B_3_, and 3D (Figure 2, Figure 3, Figure 4, Figure 5 and Figure 6). Minority recombinants also included breakpoints in region 3C (Figure 3 and Figure 5).

### 2.3. Recombinant Breakpoint Analysis

The locations of interserotypic recombinant breakpoints were analyzed. The mechanism of recombination is thought to be enabled by the RdRp (encoded by 3D) switching templates due to high regional homology between the donor and acceptor templates [29]. In order to assess sites of recombination, genomic locations of chimeric reads (the midpoints of which approximate breakpoints) were correlated with average site-wise FMDV-A:FMDV-O parental sequence homology within a 35-nt sliding window. A calculated Spearman’s correlation coefficient (𝜌) of 0.64 (*p* < 0.0001) suggested that sequence homology between the parent genomes was important in recombination. RDP4 software version 4.101 [25,30] was used to identify recombination hotspots which were compared to hotspots identified in a dataset of 767 FMDV field-derived polyprotein coding sequences (CDS) [27]. Breakpoint distribution counts identified in the 767 FMDV CDS dataset within a 35-nt sliding window were extracted and compared to those observed in recombinants within the present study. Spearman’s 𝜌 of 0.153 (*p* < 0.0001) suggested that some of the breakpoints identified in the current study may be classified as hotspots.

### 2.4. Within-Host Evolution: Genomic Substitution Rates

In order to assess whether coinfection affected virus evolution and to evaluate evolution of the recombinant viruses, nucleotide substitution rates of viruses isolated from OPF samples were measured. Rates were estimated based on consensus level changes, calculated per nucleotide per day across the FMDV polyprotein CDS. Rates were also calculated for the 2A–3D coding regions when comparing non-recombinant FMDV-A genomes to each other, or to recombinants. Comparison of the FMDV-A inoculum sequence to the FMDV-A-derived segments of each animal’s final VI-positive OPF (45 dpi_A_/24 dpi_O_–56 dpi_A_/45 dpi_O_) provided an indication of the genomic change detected over the course of the study (Table 2). Additionally, comparison of the final FMDV-A sequence OPF obtained before FMDV-O inoculation (17 or 31 dpi_A_) to the FMDV-A-derived segments of each animal’s final VI-positive OPF (45 dpi_A_/24 dpi_O_–56 dpi_A_/45 dpi_O_) provided an indication of the genomic change that took place after coinfection (Table 3).

The average number of substitutions/nucleotide/day (s/n/d) in cattle with persistent serotype A infections that had no evidence of recombinant viruses (#1931 and #1939) were 1.04 × 10^−4^ for 2A–3D, and 9.45 × 10^−5^ for the full CDS regions (Table 2). In cattle with persistently infecting FMDV-A viruses that were transiently infected with recombinant viruses (#1914, #1930, and #1932), the substitution rates for the non-recombinant FMDV-A viruses averaged 7.20 × 10^−5^ and 8.83 × 10^−5^ s/n/d (Table 2). For both groups and regions, substitution rates were consistently higher (ranging from 1.24–1.70 × 10^−4^ s/n/d) during the period of coinfection than over the entire study period (Table 3). However, the sample sizes were too limited for statistical assessment.

### 2.5. Within-Host Evolution: Non-Synonymous Changes and Potential Selective Advantages

Changes in protein coding that took place over time were analyzed in viral genomes obtained from serial OPF samples. Specifically, this analysis focused on instances in which one virus replaced another as the dominant species. In comparing the FMDV-O inoculum to the FMDV-O-derived segments of succeeding recombinants, several nonsynonymous changes were identified in structural proteins (Table 4). Most notably, Ser134Cys in VP1 was identified in all recombinants. This residue lies in the GH loop, which is both the host integrin receptor binding site and part of the primary antigenic site [31]. The minority FMDV-O viruses that were present simultaneously with the recombinants also encoded this specific change, as evidenced by their lack of variation at the subconsensus level. Other nonsynonymous changes were detected, but were only present in a subset of recombinants and did not otherwise suggest any selective advantage. Additionally, the amino acid differences were analyzed between recombinants and FMDV-A viruses in instances when one virus outcompeted the other. A maximum of two amino acid changes were identified per animal, but none were shared between animals (Table 4). The recombinants identified in animal #1929 did not encode any nonsynonymous differences in FMDV-A-derived segments. The sole virus isolated from animal #1938 OPF encoded only an Arg98Lys change within coding region 3A; nonsynonymous changes detected in the FMDV-O-derived segment were also seen in FMDV-O virus isolated in this animal.

### 2.6. In Vitro Recombinant Characterization

In order to investigate the functional characteristics of genomic changes in the chimeric viruses, one OPF sample that included an interserotypic recombinant was chosen for investigation of plaque morphology, growth rate, and specific infectivity in cell culture. The 35 dpi_A_/14 dpi_O_ #1914 sample included a dominant (>99%) interserotypic recombinant composed of an FMDV-O genome from the 5′UTR through a portion of the 2C coding region and an FMDV-A-derived genome thereafter (breakpoint ~4560, Figure 2). This recombinant was compared to each inoculum virus and to the 49 dpi_A_/28 dpi_O_ OPF sample from animal #1914. This 49 dpi_A_/28 dpi_O_ #1914 sample included only non-recombinant FMDV-A virus; this isolate was chosen because it ultimately displaced the recombinant in this animal.

**Figure 7:**
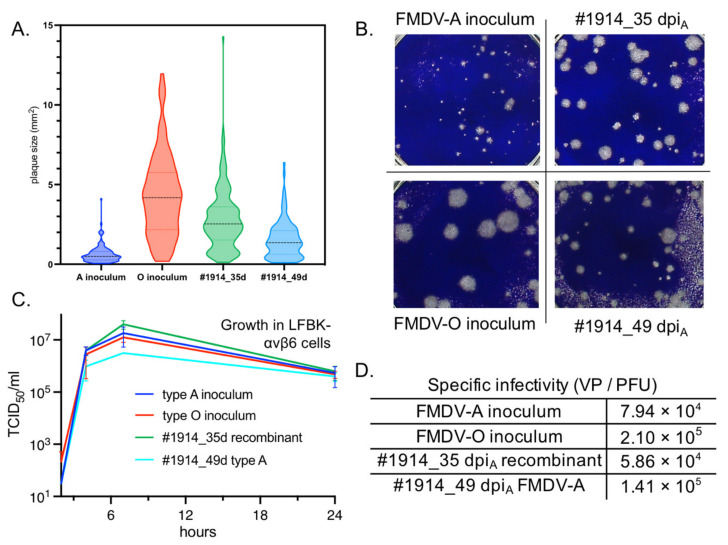
In vitro characterization of recombinant FMDV. FMDV isolated from oropharyngeal fluid sampled from animal #1914 at 35 dpi_A_/14 dpi_O_ (#1914_35d in plots) was compared to serotypes A and O inoculum viruses and non-recombinant FMDV-A virus isolated from animal #1914 at 49 dpi_A_/28 dpi_O_ (#1914_49d in plots). (**A**) Violin plots of plaque sizes measured after 24 h growth in LFBK-αvβ6 cells. Black and colored dotted lines indicate median size and quartiles, respectively. (**B**) Sample images of plaque morphology. (**C**) Growth rates of plaque-purified isolates, grown in LFBK-αvβ6 cells. (**D**) Sucrose-gradient-spectrography measures of specific infectivity (viral particle (VP) per plaque-forming unit (PFU) of plaque-purified viruses.

Samples were serially diluted and incubated for 24 h on LFBK-αvβ6 cells [32]. Plaques were stained and measured using the ViralPlaque macro in ImageJ software version 1.53 [33] (Figure 7A,B). The average plaque size of the 35 dpi_A_/14 dpi_O_ OPF sample (~99.8% recombinant) was 2.8 mm^2^. This was substantially larger than the average for the FMDV-A inoculum, 0.6 mm^2^, and smaller than FMDV-O virus plaques, 4.4 mm^2^. Interestingly, the 49 dpi_A_/28 dpi_O_ plaques (FMDV-A at study end) averaged 1.5 mm^2^, more than twice the size of the FMDV-A inoculum plaque average.

The growth rates of plaque-purified samples, confirmed by Illumina sequencing to represent the intended viruses, were similar in LFBK-αvβ6 cells (Figure 7C). Plaque-purified samples were concentrated through polyethylene glycol (PEG) precipitation, titrated and run through a sucrose density gradient with OD_260_ peak absorbances measured per fraction. Specific infectivity measures, defined as the ratio of viral particles to plaque forming units (VP/PFU) were 7.94 × 10^4^ for the FMDV-A inoculum, 2.10 × 10^5^ for the FMDV-O inoculum, 5.86 × 10^4^ for the 35 dpi_A_/14 dpi_O_ #1914 recombinant, and 1.41 × 10^5^ for the 49 dpi_A_/28 dpi_O_ #1914 non-recombinant FMDV-A virus (Figure 7D).

## 3. Discussion

It is well-established that recombination is one of the major drivers of virus evolution. Recombination of FMDV has been characterized in tissue culture systems and has been documented retrospectively in field samples. However, recombination of FMDV has never previously been documented and characterized in a natural host species under controlled laboratory conditions. The current study demonstrated that FMDV recombination occurs in the upper respiratory tract of a substantial quantity (42%) of persistently infected cattle that were superinfected with a heterologous virus strain. By contrast, within the same studies, recombinant viruses were not detected in any samples from vesicle lesions [22]. Given that dual infections with multiple FMDV strains are known to occur under natural conditions [1,2], these findings suggest that superinfection of carrier animals should be considered as a source of recombinant FMDVs detected in retrospective field studies.

Virus populations present in OPF samples were found to be highly diverse. This was particularly true of those sampled relatively early following FMDV-O superinfection (Figure 2, Figure 3, Figure 4 and Figure 5). Those samples commonly included non-recombinant serotypes A and O as well as interserotypic recombinants (Figure 2, Figure 4 and Figure 5). When recombinant viruses were detected, they always became the dominant species for at least one sampling date; in one animal, the recombinant remained the dominant virus in OPF until the end of the study. Additional recombinant viruses were also regularly identified at minority levels. Those minority species included different breakpoints from the predominant recombinants. In some cases, the minorities became the dominant virus in subsequent OPF samples. In others, they appeared to remain at low levels. While it was not possible to estimate the full extent of recombination that these populations suggest, it is clearly a common event.

Consistent with the complexity of OPF virus populations, substitution rates were higher during the periods of superinfection compared to the periods of single virus infection. This elevated rate of within-host evolution during FMDV co-infection suggests that distinct immune mechanisms and/or selective pressures may exist during co-infection when compared to single strain infection.

Findings from in vitro assays did not indicate significant growth differences between the parental viruses and a selected interserotypic recombinant. The specific infectivity of this recombinant had a lower ratio of viral particles to plaque forming units than its parents or its successor. That specific measurement is impacted by multiple aspects of the virus infection cycle, and thus does not necessarily indicate that the recombinant is more fit overall. Nevertheless, that finding suggests that packaging an interserotypic recombinant FMDV genome into an FMDV-O capsid does not incur a fitness loss. This is consistent with a recent investigation of cell culture coinfection which demonstrated trans-encapsidation of serotypes FMDV-Asia 1 and FMDV-O genomes in heterologous capsids [34].

All recombinant viruses detected in these studies encoded FMDV-O capsids. Serotypes A and O are antigenically distinct, meaning that acquired immunity against one does not protect against infection with the other serotype. Thus, the animals’ capacity to neutralize FMDV-O capsids is presumed to have been weaker directly following superinfection with that serotype, while the adaptive response to FMDV-A capsids was already well-established at that time [22]. This concept largely explains the emergence of recombinant viruses encoding FMDV-O capsids in the present study. However, the mechanistic advantage conferred by the FMDV-O capsid combined with FMDV-A-derived nonstructural coding regions remains undetermined. Similarly, it is undetermined why, in some cases, these recombinants emerged to dominance but were out-competed by non-recombinant viruses. Studies of human pathogens causing chronic infection such as HIV-1 and human cytomegalovirus among others have identified recombination between distinct viruses following superinfection [35,36,37]. Further, these studies implicate host immune evasion as a main driver of recombinant virus emergence. While the present findings suggest similar phenomena, future investigations into the FMDV acute phase and persistent phase viral populations are required to fully address these hypotheses.

There was no consistency in the dominance of viruses detected in final OPF samples across the 12 superinfected cattle. Amino acid analysis did not indicate any specific change related to dominance of one genome over another. Nevertheless, the success of interserotypic recombinant viruses over non-recombinant FMDV-O viruses is curious. This is because both groups encode FMDV-O capsids, presumably subject to comparable immune pressure. Analysis of nonsynonymous substitutions identified several amino acid changes in recombinants, most notably a change at the receptor binding site. However, this change was present in the non-recombinant FMDV-O genomes as well; thus, it does not suggest an advantage that was unique to recombinants. A more substantial difference between the recombinants and the non-recombinant FMDV-O viruses are the FMDV-A-derived portions of the recombinant genomes. FMDV recombination hotspots have been consistently identified to fall between the functional units of the genome, e.g., capsid, proteases, polymerase etc. [19,38]. If the (inconsistent) dominance of recombinants over non-recombinants is in part due to higher fitness, the location of recombinant breakpoints can hint at the protein coding regions that confer this advantage. Breakpoints were identified in nearly all of the nonstructural protein coding regions. Thus, the only FMDV-A-derived segment that was present in all recombinants was the C-terminal portion of 3D. Interestingly, 3D is very highly conserved, as evidenced by only two amino acid differences between the two serotypes in the affected region. It is possible that some fitness advantages may have been conferred by differences in noncoding regions that were beyond the scope of this study.

Mechanistically, when the viral polymerase switches templates during a recombination event, it has been proposed that this movement is facilitated by elevated local sequence identity and related secondary structure elements between the donor and acceptor templates [39,40,41,42]. The breakpoints identified in the present work correlated very well (Spearman’s correlation coefficient = 0.64) with pairwise sequence identity, which is consistent with the dogma that template switching may be facilitated by homology. In comparing the breakpoints identified in the present work with those identified across a dataset of 767 field-derived FMDV sequences, there was a weak but positive correlation (𝜌 = 0.153). This suggests that some of the breakpoints observed in the current investigation may not be as common or consistent in recombinants that have been isolated in the field. Alternatively, the diverse recombinants observed in the present study may represent examples of intermediaries (snapshots) in the emergence of more stable recombinants that might become epidemiologically relevant on a longer timeline, as has been shown in picornavirus recombination mechanism research [43,44].

The mechanisms by which FMDV maintains persistent infection are incompletely understood [7]. Studies have suggested down-regulation of the cellular immune response and apoptotic pathways in nasopharyngeal epithelium in association with persistent FMDV infection [13,45,46,47]. Additionally, it has been demonstrated that during both primary and persistent infection, only a small subset of nasopharyngeal epithelial cells become infected with FMDV [48,49]. Further, recent work from our laboratory has revealed that during the carrier phase, contiguous specimens of nasopharyngeal epithelium contain foci of distinct FMDV variants. It is likely that interplay of this multifocal viral clonality combined with regionally variable mucosal immune responses may contribute to variant emergence and dominance more than fitness advantages being conferred by specific substitutions or segments. Further investigation of regionally specific host responses and FMDV populations in these tissues is needed to investigate the complex interplay of viral and host cellular factors involved in this process.

In all animals in which recombinants were detected, chimeric viruses were found to be the dominant virus in the first OPF sample obtained following FMDV-O infection, corresponding to 10–14 days after superinfection. Because recombinants were already dominant at these sampling time points, it is likely that these viruses were common, if not dominant, at earlier timepoints as well. However, the precise timing of onset of detection of recombination was not determined herein. Importantly, those earlier time points are when FMDV transmission is more likely during single-virus infections [28]. This is relevant to the concept that FMDV carriers are not believed to be significant sources of transmission [50,51].

The samples used in the current study were derived from persistently infected carrier animals that were superinfected with a heterologous virus. These animals thereby represent a distinct category as they were both persistently and acutely infected, regardless of their clinical status. Coinfection is not uncommon in FMD-endemic regions where multiple serotypes are in circulation [1,2,3,4] and superinfection of FMDV carriers comprises a subset of these dual infections. However, there are infinite permutations of the timing and strain involvement which could affect superinfection under natural conditions. The two timepoints and strains of superinfection investigated in the present work are plausible representatives of this diverse potential variability, however the output described herein should not be interpreted as paradigmatic for all potential iterations. FMDV coinfection separated by fewer than 21, or more than 35 days could induce different frequencies or patterns of recombination than were observed in the current investigation. Furthermore, different viral strains and host genetic backgrounds could also affect such output. Ultimately, greater understanding of the role of recombination in FMDV evolution and epidemiology will only be achieved through a combination of additional laboratory-based investigations and field-based longitudinal studies in FMDV-endemic countries.

We have previously characterized the neoteric (early) subclinical phase of FMDV infection as distinct from the more commonly recognized persistent phase of infection based on higher quantities of virus detectable in oronasal secretions. This neoteric subclinical shedding of FMDV has an increased probability of transmission compared to persistently infected carriers that are not superinfected [12,13,51]. Importantly, the period during which recombinant viruses were most common in these cattle occurred within the neoteric phase. Therefore, we hypothesize that transmission of novel recombinant FMDV in a carrier-superinfection scenario is more likely than in a typical persistent infection. However, the transmissibility of emergent viruses from superinfected carriers remains to be demonstrated, and this is a current focus of work in our laboratory. If proven experimentally, this relationship between FMDV persistence, superinfection, recombination, and transmission would have substantial implications for FMD epidemiology and control measures.

## 4. Materials and Methods

### 4.1. In Vivo Study and Sample Background

Virus samples used for the current investigation were derived from a previously published study investigating the pathogenesis of FMDV coinfection in cattle, using two bovine-derived and well-characterized FMDV isolates of strains A24 Cruzeiro and O1 Manisa [22]. The 12 cattle from which samples used for the current investigation were derived were first infected by intra-nasopharyngeal deposition of 10^6^ TCID_50_ of FMDV A24, after which all animals developed clinical FMD of expected severity. After either 21 (eight animals) or 35 (four animals) days, the animals were superinfected with FMDV O1 Manisa using the same titer and route. Details of the pathogenesis across these studies have been previously published [22]. Oropharyngeal fluid samples were taken from all animals 4–7 days prior to serotype O inoculation, all of which were confirmed to be positive for infectious FMDV. OPF sampling was not performed during acute infection, but restarted at 10–14 days following serotype O inoculation.

### 4.2. Illumina Sequencing and Assembly

Deep-sequencing of virus isolates analyzed in the present work was performed on the NextSeq 550 (Illumina) from OPF samples passaged once in LFBK-αvβ6 cells [32]. FMDV full-length genome sequencing was performed as previously described [22]. In short, FMDV RNA was extracted from cell culture supernatant using the MagMAX RNA Isolation Kit (ThermoFisher), reverse-transcribed using Superscript II (Invitrogen) using random hexameric primers, followed by Nextera XT (Illumina) library construction. All samples were sequenced in duplicate at minimum.

CLC Genomics Workbench v. 20–v. 21 was used to filter reads for quality and length and competitively assemble them to each of the two reference genomes corresponding to the two inocula, A24 Cruzeiro (GenBank #AY593768) and O1 Manisa (#AY593823). The identification of recombinant genomes was initially based on inconsistencies in read mapping, but later confirmed by Sanger sequencing amplicons created with breakpoint-crossing primers, de novo assembly, resequencing, sequencing of un-passaged samples, chimeric read analysis and haplotype reconstruction; all methods covered in brief here are detailed in [22].

### 4.3. Breakpoint Association Analysis

Breakpoints were identified both at the consensus and subconsensus level through a combination of significant reference genome coverage changes, chimeric read identification and haplotype reconstruction. A subset of samples from each animal were also Sanger sequenced as noted above. Chimeric reads were mapped to the CDS of each reference genome and the site-wise coverage values were extracted. The product of site-wise chimeric read coverage between each reference was calculated such that putative chimeric reads that only mapped to one parental genome were discounted. This data set was then compared to breakpoints predicted among a large, 769 FMDV CDS data set (767 with the inoculum viruses removed) curated and published by Aiewsakun, Pamornchainavakul and Inchaisri [27] using the Breakpoint Distribution Plot (35-nt window) [25] function in RDP4 software [30]. Breakpoints were also compared to site-wise homology between the two parental genomes, FMDV-A and FMDV-O. Spearman’s correlation coefficient was calculated in Prism 9.2 (GraphPad Software).

### 4.4. Haplotype Reconstruction

FMDV populations are comprised of numerous genomic variants, regularly called a mutant swarm or quasispecies. For FMDV in particular, variant virus genomes within FMDV populations have been found to fall into clades or haplogroups that can persist over time [52,53]. In order to computationally reconstruct the major component viral haplotypes, the software CliqueSNV, version 1.41 [54] was implemented. Reads mapped in CLC Genomics Workbench to the 2A–3D genomic region of the FMDV-O reference genome were extracted to BAM format and run in CliqueSNV with the parameters -t (20–100) and -tf (0.0001–0.005), depending on average coverage and estimated SNV frequencies (as estimated in CLC). In addition, haplotypes were also analyzed with the reconstruction program QuasiRecomb [55] with the -conservative flag.

### 4.5. In Vitro Virus Analysis

Each inoculum, the recombinant virus identified in the 35 dpi_A_/14 dpi_O_ OPF sample from animal #1914, and the FMDV-A (non-recombinant) virus identified in the 49 dpi_A_/28 dpi_O_ sample from animal #1914 were amplified on LFBK-αvβ6 cells for 24 h [32]. Next, each sample was serially diluted and incubated on LFBK-αvβ6 cells for 24 h. Plates were stained and viral plaques were measured using the ViralPlaque macro [33] in ImageJ v. 1.53 [56].

To ensure genetic homogeneity for subsequent analyses, plaques were isolated for each inoculum as well as both the 35 dpi_A_/14 dpi_O_ and 49 dpi_A_/28 dpi_O_ OPFs from animal #1914. Each sample was serially diluted and plated on LFBK-αvβ6 cells in 6-well cell culture plates (low-MP agar, 37 °C, 4.9% CO_2_). Discrete plaques were picked, transferred to 500 μL PBS and stored at −70 °C. Thawed plaques were then incubated on LFBK-αvβ6 cells for 24–48 h (until cytopathic effect was observed) and stored at −70 °C. Homogeneity of each isolate was confirmed through targeted RT-PCR as well as Illumina sequencing as described above. Each virus was then amplified in LFBK-αvβ6 cells in 2 L roller bottles. Lastly, each virus was concentrated using polyethylene glycol precipitation and titrated.

Growth rates of each virus were measured in LFBK-αvβ6 cells, with aliquots titrated at 1, 2, 4, 7, and 24 h. Growth was below the level of detection for 1 and 2 h timepoints except for FMDV-O which averaged 2.17 × 10^2^ TCID_50_/mL at 2 h. Specific infectivity was measured via sucrose gradient diffusion followed by spectrographic analysis [57,58]; Samples were titrated by plaque assay and OD_260_ was measured for each virus within the same gradient fraction corresponding to the encapsidated virus absorbance peak.

## Figures and Tables

**Table 1:** Total reads mapped from oropharyngeal fluid samples to FMDV-A and FMDV-O reference sequences.

Superinfected with FMVD-O at 21 Days Post-FMDV-A Infection											
	**Days post FMDV-A Infection:**	31	35	38	42	43	45	46	49	52	56
**Cattle ID**	**Days post FMDV-O Infection:**	10	14	17	21	22	24	25	28	31	35
#1913	FMDV-A coverage	na	nd	nd	na	nd	nd	na	nd	na	na
	FMDV-O coverage	na	3,184,243	3,003,584	na	2,498,066	2,815,675	na	408,851	na	na
#1914	FMDV-A coverage	na	1,466,016 **	1,108,314 **	na	na	1,336,215 *	na	632,293	na	na
	FMDV-O coverage	na	2,038,351 **	1,585,477 **	na	na	78,772 *	na	nd	na	na
#1915	FMDV-A coverage	na	nd	nd	na	nd	nd	nd	na	na	na
	FMDV-O coverage	na	3,286,594	2,441,424	na	3,255,000	2,205,781	231,427	na	na	na
#1916	FMDV-A coverage	na	509,327	nd	na	nd	nd	nd	na	na	na
	FMDV-O coverage	na	nd	2,114,614	na	2,257,357	2,381,671	954,136	na	na	na
#1929	FMDV-A coverage	651,366 **	446,848 **	475,485 **	248,902 **	na	430,237 **	na	409,467 **	393,012 **	354,532 **
	FMDV-O coverage	1,705,723 **	1,591,709 **	1,736,492 **	1,356,927 **	na	2,349,218 **	na	2,390,024 **	2,290,827 **	2,175,387 **
#1930	FMDV-A coverage	323,622 **	301,255 **	924,836 *	623,192 **	na	1,212,858	na	1,349,957	2,439,414	1,336,192
	FMDV-O coverage	1,417,422 **	539,485 **	233,335 *	496,749 **	na	nd	na	nd	nd	nd
#1931	FMDV-A coverage	1,138,949	1,831,884	1,519,394	2,273,947	na	728,656	na	2,711,542	849,330	1,160,417
	FMDV-O coverage	nd	nd	nd	nd	na	nd	na	nd	nd	nd
#1932	FMDV-A coverage	287,724 **	483,755 **	992,598 **	1,017,763 **	na	na	na	1,336,430	2,474,777	2,520,676
	FMDV-O coverage	568,194 **	585,192 **	1,627,739 **	1,152,934 **	na	na	na	nd	nd	nd
**Superinfected with FMVD-O at 35 Days post-FMDV-A Infection**											
	**Days post FMDV-A Infection**	45	49	52	56	59	63	66	70		
**Cattle ID**	**Days post FMDV-O Infection**	10	14	17	21	24	28	31	35		
#1938	FMDV-A coverage	2,513,886 **	neg	neg	neg	neg	neg	neg	neg		
	FMDV-O coverage	2,613,373 **	neg	neg	neg	neg	neg	neg	neg		
#1939	FMDV-A coverage	2,188,164	4,152,638	neg	2,501,929	neg	neg	neg	neg		
	FMDV-O coverage	nd	nd	neg	nd	neg	neg	neg	neg		

na—sample not acquired; nd—specific virus not detected; neg—negative for infectious virus; *—inter-serotypic recombinant detected; **—inter-serotypic recombinant detected and dominant.

**Table 2:** Substitution rates for full study period.

					Total Substitutions from Day 0 (FMDV-A Inoculum Consensus) through Final OPF Sample					
					Coding Region 2A–3D			CDS		
Cattle ID	Final Virus	Was a Recombinant Ever Detected?	Last OPF Sample dpi_A_ before FMDV-O Inoculation	Final Virus dpi_A_	Number of FMDV-A Substitutions	Number of FMDV-A Segment Substitutions per nt	Subs/nt/Day	Number of FMDV-A Substitutions	Number of FMDV-A Segment Substitutions per nt	Subs/nt/Day
#1914	FMDV-A	yes	17	49	19	0.00453	9.24 × 10^−5^	40	0.00572	1.17 × 10^−4^
#1930	FMDV-A	yes	17	56	12	0.00286	5.11 × 10^−5^	27	0.00386	6.89 × 10^−5^
#1932	FMDV-A	yes	17	56	17	0.00406	7.25 × 10^−5^	31	0.00443	7.91 × 10^−5^
						average:	7.20 × 10^−5^		average:	8.83 × 10^−5^
#1929	Recombinant	yes	17	56	2	0.00136	2.43 × 10^−5^	na	na	na
#1938	Recombinant	yes	31	45	16	0.00391	8.69 × 10^−5^	na	na	na
						average:	5.56 × 10^−5^			
#1931	FMDV-A	no	17	56	26	0.0062	1.11 × 10^−4^	37	0.00529	9.45 × 10^−5^
#1939	FMDV-A	no	31	56	23	0.00549	9.80 × 10^−5^	37	0.00529	9.45 × 10^−5^
						average:	1.04 × 10^−4^		average:	9.45 × 10^−5^

Abbreviations: OPF—oropharyngeal fluid; dpi_A_—days post FMDV-A infection; dpi_O_—days since FMDV-O infection; subs—substitutions; nt—nucleotides; na—not applicable.

**Table 3:** Substitution rates for coinfection period.

					Total FMDV-A Substitutions from Last OPF Sample (before FMDV-O Infection) through Final OPF Sample					
					Coding Region 2A–3D			CDS		
Cattle ID	Final Virus	Was a Recombinant Ever Detected?	Last OPF Sample dpi_A_ before FMDV-O Inoculation	Final Virus dpi_A_	Number of FMDV-A Substitutions	Number of FMDV-A Segment Substitutions per nt	Subs/nt/Day	Number of FMDV-A Substitutions	Number of FMDV-A Segment Substitutions per nt	Subs/nt/Day
#1914	FMDV-A	yes	17	49	17	0.00406	1.27 × 10^−4^	30	0.00429	1.34 × 10^−4^
#1930	FMDV-A	yes	17	56	19	0.00453	1.16 × 10^−4^	42	0.00600	1.54 × 10^−4^
#1932	FMDV-A	yes	17	56	21	0.00501	1.28 × 10^−4^	40	0.00572	1.47 × 10^−4^
						average:	1.24 × 10^−4^		average:	1.45 × 10^−4^
#1929	Recombinant	yes	17	56	1	0.00068	1.74 × 10^−5^	na	na	na
#1938	Recombinant	yes	31	45	12	0.00293	2.09 × 10^−4^	na	na	na
						average:	1.13 × 10^−4^			
#1931	FMDV-A	no	17	56	26	0.00620	1.59 × 10^−4^	37	0.00529	1.36 × 10^−4^
#1939	FMDV-A	no	31	56	19	0.00453	1.81 × 10^−4^	25	0.00357	1.43 × 10^−4^
						average:	1.70 × 10^−4^		average:	1.39 × 10^−4^

Abbreviations: OPF—oropharyngeal fluid; dpi_A_—days post FMDV-A infection; dpi_O_—days since FMDV-O infection; subs—substitutions; nt—nucleotides; na—not applicable.

**Table 4:** Nonsynonymous changes in virus successions.

Cattle ID	Virus Succession	Coding Region(s) and Change(s)	Virus Succession	Coding Region(s) and Change(s)
#1914	FMDV-O > Recombinant	VP1: Ser134Cys ^†^	Rec > FMDV-A	3D: Cys76Arg
#1929	FMDV-O > Recombinant	VP3: Ala75Thr, VP1: Ser134Cys ^†^, Ile194Val	FMDV-A > Rec *	none
#1930	FMDV-O > Recombinant	VP1: Ser134Cys ^†^	Rec > FMDV-A	3A: Val134Ala ^‡^, 3D: Cys174Arg ^‡^
#1932	na (all viruses present until 45 dpi_A_)	VP2: Met182Val, VP1: Ser134Cys ^†^	Rec and FMDV-O > FMDV-A	none ^‡^
#1938	FMDV-O > Recombinant	Leader: Leu80Phe, VP2: Met182Val, VP1: Ser134Cys ^†^	FMDV-A > Rec *	3A: Arg96Lys

* no non-recombinant FMDV-A detected in OPF after second infection; ^†^ polymorphic in inoculum; ^‡^ high-level FMDV-A in all OPFs means this is unresolved.

## Data Availability

Sequence Read Archive (SRA) files are freely available at ncbi.nlm.nih.gov (NCBI) under BioProject no. PRJNA662932.

## References

[1] Farooq U., Ahmed Z., Naeem K., Bertram M., Brito B., Stenfeldt C., Pauszek S.J., LaRocco M., Rodriguez L., Arzt J. (2018). Characterization of naturally occurring, new and persistent subclinical foot-and-mouth disease virus infection in vaccinated Asian buffalo in Islamabad Capital Territory, Pakistan. Transbound. Emerg. Dis..

[2] Omondi G.P., Gakuya F., Arzt J., Sangula A., Hartwig E., Pauszek S., Smoliga G., Brito B., Perez A., Obanda V. (2020). The role of African buffalo in the epidemiology of foot-and-mouth disease in sympatric cattle and buffalo populations in Kenya. Transbound. Emerg. Dis..

[3] Al-Hosary A.A., Kandeil A., El-Taweel A.N., Nordengrahn A., Merza M., Badra R., Kayali G., Ali M.A. (2019). Co-infection with different serotypes of FMDV in vaccinated cattle in Southern Egypt. Virus Genes.

[4] Palinski R., Brito B., Arzt J. (2022). Foot-and-mouth disease virus population diversity in naturally infected African buffalo in Kenya co-infected with SAT1 and SAT2 serotypes. Viruses.

[5] Arzt J., Juleff N., Zhang Z., Rodriguez L.L. (2011). The pathogenesis of foot-and-mouth disease I: Viral pathways in cattle. Transbound. Emerg. Dis..

[6] Bertram M.R., Yadav S., Stenfeldt C., Delgado A., Arzt J. (2020). Extinction Dynamics of the Foot-and-Mouth Disease Virus Carrier State Under Natural Conditions. Front. Vet. Sci..

[7] Stenfeldt C., Arzt J. (2020). The Carrier Conundrum; A Review of Recent Advances and Persistent Gaps Regarding the Carrier State of Foot-and-Mouth Disease Virus. Pathogens.

[8] Tenzin, Dekker A., Vernooij H., Bouma A., Stegeman A. (2008). Rate of foot-and-mouth disease virus transmission by carriers quantified from experimental data. Risk Anal..

[9] Sutmoller P., Gaggero A. (1965). Foot-and mouth diseases carriers. Vet. Rec..

[10] Van Bekkum J.G., Frenkel H.S., Frederiks H.H.J., Frenkel S. (1959). Observations on the carrier state of cattle exposed to foot-and-mouth disease virus. Tijdschr. Diergeneeskd..

[11] Stenfeldt C., Eschbaumer M., Pacheco J.M., Rekant S.I., Rodriguez L.L., Arzt J. (2015). Pathogenesis of Primary Foot-and-Mouth Disease Virus Infection in the Nasopharynx of Vaccinated and Non-Vaccinated Cattle. PLoS ONE.

[12] Stenfeldt C., Lohse L., Belsham G.J. (2013). The comparative utility of oral swabs and probang samples for detection of foot-and-mouth disease virus infection in cattle and pigs. Vet. Microbiol..

[13] Stenfeldt C., Eschbaumer M., Rekant S.I., Pacheco J.M., Smoliga G.R., Hartwig E.J., Rodriguez L.L., Arzt J. (2016). The Foot-and-Mouth Disease Carrier State Divergence in Cattle. J. Virol..

[14] Pringle C.R. (1968). Recombination between conditional lethal mutants within a strain of foot-and-mouth disease virus. J. Gen. Virol..

[15] Pringle C.R. (1965). Evidence of Genetic Recombination in Foot-and-Mouth Disease Virus. Virology.

[16] King A.M., McCahon D., Slade W.R., Newman J.W. (1982). Biochemical evidence of recombination within the unsegmented RNA genome of aphthovirus. J. Virol..

[17] King A.M., McCahon D., Saunders K., Newman J.W., Slade W.R. (1985). Multiple sites of recombination within the RNA genome of foot-and-mouth disease virus. Virus Res..

[18] Lewis-Rogers N., McClellan D.A., Crandall K.A. (2008). The evolution of foot-and-mouth disease virus: Impacts of recombination and selection. Infect. Genet. Evol..

[19] Brito B., Pauszek S.J., Hartwig E.J., Smoliga G.R., Vu L.T., Dong P.V., Stenfeldt C., Rodriguez L.L., King D.P., Knowles N.J. (2018). A traditional evolutionary history of foot-and-mouth disease viruses in Southeast Asia challenged by analyses of non-structural protein coding sequences. Sci. Rep..

[20] Jamal S.M., Ferrari G., Ahmed S., Normann P., Belsham G.J. (2011). Molecular characterization of serotype Asia-1 foot-and-mouth disease viruses in Pakistan and Afghanistan; emergence of a new genetic Group and evidence for a novel recombinant virus. Infect. Genet. Evol..

[21] Jamal S.M., Nazem Shirazi M.H., Ozyoruk F., Parlak U., Normann P., Belsham G.J. (2020). Evidence for multiple recombination events within foot-and-mouth disease viruses circulating in West Eurasia. Transbound. Emerg. Dis..

[22] Arzt J., Fish I.H., Bertram M.R., Smoliga G.R., Hartwig E.J., Pauszek S.J., Holinka-Patterson L., Diaz-San Segundo F.C., Sitt T., Rieder E. (2021). Simultaneous and staggered foot-and-mouth disease virus coinfection of cattle. J. Virol..

[23] Kirkegaard K., Baltimore D. (1986). The mechanism of RNA recombination in poliovirus. Cell.

[24] Simon-Loriere E., Holmes E.C. (2011). Why do RNA viruses recombine?. Nat. Rev. Microbiol..

[25] Heath L., van der Walt E., Varsani A., Martin D.P. (2006). Recombination patterns in aphthoviruses mirror those found in other picornaviruses. J. Virol..

[26] Simmonds P. (2006). Recombination and selection in the evolution of picornaviruses and other Mammalian positive-stranded RNA viruses. J. Virol..

[27] Aiewsakun P., Pamornchainavakul N., Inchaisri C. (2020). Early origin and global colonisation of foot-and-mouth disease virus. Sci. Rep..

[28] Yadav S., Stenfeldt C., Branan M.A., Moreno-Torres K.I., Holmstrom L.K., Delgado A.H., Arzt J. (2019). Parameterization of the Durations of Phases of Foot-And-Mouth Disease in Cattle. Front. Vet. Sci..

[29] Woodman A., Lee K.M., Janissen R., Gong Y.N., Dekker N.H., Shih S.R., Cameron C.E. (2019). Predicting Intraserotypic Recombination in Enterovirus 71. J. Virol..

[30] Martin D.P., Lemey P., Lott M., Moulton V., Posada D., Lefeuvre P. (2010). RDP3: A flexible and fast computer program for analyzing recombination. Bioinformatics.

[31] Kotecha A., Wang Q., Dong X., Ilca S.L., Ondiviela M., Zihe R., Seago J., Charleston B., Fry E.E., Abrescia N.G.A. (2017). Rules of engagement between alphavbeta6 integrin and foot-and-mouth disease virus. Nat. Commun..

[32] LaRocco M., Krug P.W., Kramer E., Ahmed Z., Pacheco J.M., Duque H., Baxt B., Rodriguez L.L. (2013). A continuous bovine kidney cell line constitutively expressing bovine alphavbeta6 integrin has increased susceptibility to foot-and-mouth disease virus. J. Clin. Microbiol..

[33] Cacciabue M., Curra A., Gismondi M.I. (2019). ViralPlaque: A Fiji macro for automated assessment of viral plaque statistics. PeerJ.

[34] Childs K., Juleff N., Moffat K., Seago J. (2021). Demonstration of Co-Infection and Trans-Encapsidation of Viral RNA In Vitro Using Epitope-Tagged Foot-and-Mouth Disease Viruses. Viruses.

[35] Streeck H., Li B., Poon A.F., Schneidewind A., Gladden A.D., Power K.A., Daskalakis D., Bazner S., Zuniga R., Brander C. (2008). Immune-driven recombination and loss of control after HIV superinfection. J. Exp. Med..

[36] Koning F.A., Badhan A., Shaw S., Fisher M., Mbisa J.L., Cane P.A. (2013). Dynamics of HIV type 1 recombination following superinfection. AIDS Res. Hum. Retrovir..

[37] Cudini J., Roy S., Houldcroft C.J., Bryant J.M., Depledge D.P., Tutill H., Veys P., Williams R., Worth A.J.J., Tamuri A.U. (2019). Human cytomegalovirus haplotype reconstruction reveals high diversity due to superinfection and evidence of within-host recombination. Proc. Natl. Acad. Sci. USA.

[38] Jackson A.L., O’Neill H., Maree F., Blignaut B., Carrillo C., Rodriguez L., Haydon D.T. (2007). Mosaic structure of foot-and-mouth disease virus genomes. J. Gen. Virol..

[39] Romanova L.I., Blinov V.M., Tolskaya E.A., Viktorova E.G., Kolesnikova M.S., Guseva E.A., Agol V.I. (1986). The primary structure of crossover regions of intertypic poliovirus recombinants: A model of recombination between RNA genomes. Virology.

[40] Cascone P.J., Haydar T.F., Simon A.E. (1993). Sequences and structures required for recombination between virus-associated RNAs. Science.

[41] Wilson V., Taylor P., Desselberger U. (1988). Crossover regions in foot-and-mouth disease virus (FMDV) recombinants correspond to regions of high local secondary structure. Arch. Virol..

[42] Lasecka-Dykes L., Tulloch F., Simmonds P., Luke G.A., Ribeca P., Gold S., Knowles N.J., Wright C.F., Wadsworth J., Azhar M. (2021). Mutagenesis Mapping of RNA Structures within the Foot-and-Mouth Disease Virus Genome Reveals Functional Elements Localized in the Polymerase (3D(pol))-Encoding Region. mSphere.

[43] Lowry K., Woodman A., Cook J., Evans D.J. (2014). Recombination in enteroviruses is a biphasic replicative process involving the generation of greater-than genome length ‘imprecise’ intermediates. PLoS Pathog..

[44] Bentley K., Alnaji F.G., Woodford L., Jones S., Woodman A., Evans D.J. (2021). Imprecise recombinant viruses evolve via a fitness-driven, iterative process of polymerase template-switching events. PLoS Pathog..

[45] Eschbaumer M., Stenfeldt C., Smoliga G.R., Pacheco J.M., Rodriguez L.L., Li R.W., Zhu J., Arzt J. (2016). Transcriptomic Analysis of Persistent Infection with Foot-and-Mouth Disease Virus in Cattle Suggests Impairment of Apoptosis and Cell-Mediated Immunity in the Nasopharynx. PLoS ONE.

[46] Stenfeldt C., Eschbaumer M., Smoliga G.R., Rodriguez L.L., Zhu J., Arzt J. (2017). Clearance of a persistent picornavirus infection is associated with enhanced pro-apoptotic and cellular immune responses. Sci. Rep..

[47] Zhu Z., Li C., Du X., Wang G., Cao W., Yang F., Feng H., Zhang X., Shi Z., Liu H. (2017). Foot-and-mouth disease virus infection inhibits LGP2 protein expression to exaggerate inflammatory response and promote viral replication. Cell Death Dis..

[48] Arzt J., Pacheco J.M., Rodriguez L.L. (2010). The early pathogenesis of foot-and-mouth disease in cattle after aerosol inoculation. Identification of the nasopharynx as the primary site of infection. Vet. Pathol..

[49] Stenfeldt C., Hartwig E.J., Smoliga G.R., Palinski R., Silva E.B., Bertram M.R., Fish I.H., Pauszek S.J., Arzt J. (2018). Contact Challenge of Cattle with Foot-and-Mouth Disease Virus Validates the Role of the Nasopharyngeal Epithelium as the Site of Primary and Persistent Infection. mSphere.

[50] Bertram M.R., Vu L.T., Pauszek S.J., Brito B.P., Hartwig E.J., Smoliga G.R., Hoang B.H., Phuong N.T., Stenfeldt C., Fish I.H. (2018). Lack of Transmission of Foot-and-Mouth Disease Virus From Persistently Infected Cattle to Naive Cattle Under Field Conditions in Vietnam. Front. Vet. Sci..

[51] Parthiban A.B., Mahapatra M., Gubbins S., Parida S. (2015). Virus Excretion from Foot-And-Mouth Disease Virus Carrier Cattle and Their Potential Role in Causing New Outbreaks. PLoS ONE.

[52] Arzt J., Fish I., Pauszek S.J., Johnson S.L., Chain P.S., Rai D.K., Rieder E., Goldberg T.L., Rodriguez L.L., Stenfeldt C. (2019). The evolution of a super-swarm of foot-and-mouth disease virus in cattle. PLoS ONE.

[53] Fish I., Stenfeldt C., Palinski R.M., Pauszek S.J., Arzt J. (2020). Into the Deep (Sequence) of the Foot-and-Mouth Disease Virus Gene Pool: Bottlenecks and Adaptation during Infection in Naive and Vaccinated Cattle. Pathogens.

[54] Knyazev S., Tsyvina V., Shankar A., Melnyk A., Artyomenko A., Malygina T., Porozov Y.B., Campbell E.M., Switzer W.M., Skums P. (2021). Accurate assembly of minority viral haplotypes from next-generation sequencing through efficient noise reduction. Nucleic Acids Res..

[55] Topfer A., Zagordi O., Prabhakaran S., Roth V., Halperin E., Beerenwinkel N. (2013). Probabilistic inference of viral quasispecies subject to recombination. J. Comput. Biol..

[56] Schneider C.A., Rasband W.S., Eliceiri K.W. (2012). NIH Image to ImageJ: 25 years of image analysis. Nat. Methods.

[57] Diaz-San Segundo F., Medina G.N., Ramirez-Medina E., Velazquez-Salinas L., Koster M., Grubman M.J., de los Santos T. (2016). Synonymous deoptimization of foot-and-mouth disease virus causes attenuation in vivo while inducing a strong neutralizing antibody response. J. Virol..

[58] Bachrach H.L., Trautman R., Breese S.S. (1964). Chemical Physical Properties of Virtually Pure Foot-and-Mouth Disease Virus. Am. J. Vet. Res..

